# The role of intestinal microecology in inflammatory bowel disease and colorectal cancer: A review

**DOI:** 10.1097/MD.0000000000036590

**Published:** 2023-12-22

**Authors:** Huimin Li, Kun Wang, Mengdi Hao, Yin Liu, Xiaoqing Liang, Dajin Yuan, Lei Ding

**Affiliations:** a Department of Oncology, Beijing Shijitan Hospital, Capital Medical University, Beijing, China; b Department of Oncology, Ninth School of Clinical Medicine, Peking University, Beijing, China.

**Keywords:** colorectal cancer, inflammatory bowel disease, intestinal microbiota, intestinal mucosa immune

## Abstract

Intestinal microecology is a dominant and complex microecological system in human body. Generally, intestinal microecosystem consists of normal symbiotic flora and its living environment (including intestinal epithelial tissue and intestinal mucosal immune system). Commensal flora is the core component of microecology. Both structures of intestinal mucosa and functions of immune system are essential to maintain homeostasis of intestinal microecosystem. Under normal conditions, intestinal microorganisms and intestinal mucosa coordinate with each other to promote host immunity. When certain factors in the intestine are altered, such as disruption of the intestinal barrier causing dysbiosis of the intestinal flora, the immune system of the host intestinal mucosa makes a series of responses, which leads to the development of intestinal inflammation and promotes colorectal cancer. In this review, to further understand the relationship between intestinal microecology and intestinal diseases, we systematically elaborate the composition of the intestinal mucosal immune system, analyze the relationship between intestinal flora and mucosal immune system, and the role of intestinal flora on intestinal inflammatory diseases and colorectal cancer.

## 1. Introduction

Gut microbiota plays critical roles in human health and diseases. Although microbiota was overlooked previously, this perception has been changed dramatically in 2018, as microbiota reflects complicated biological ecosystem in mutual communication with its host.^[1]^ It is well known that human intestine, hosting 3.8 × 1013 bacteria, is a huge repertoire for microbiota.^[2]^ Other microorganisms including viruses, archaea, and fungi also exist in gastrointestinal tract.^[3]^ Undoubtedly, microbiota, an extremely dense and complex ecosystem, has been recognized as an important component of host immune regulation.^[4]^ Intestinal flora is closely related to host’s immune system. In a healthy state, there is a symbiotic relationship between host and microorganisms with restricted lumen.^[3]^ Symbiotic microbiota provides nutrients and vitamins, maintains epithelial mucosal homeostasis, and protects the host from invasion by opportunistic pathogens.^[5,6]^ In addition, symbiotic microbiota is essential for intestinal structure and immune regulation.^[7,8]^ Importantly, metabolites of intestinal microbiota are conducive to host’s health.^[9]^ However, dysregulation of these interactions can lead to inflammation-related diseases.^[10,11]^ Accumulating evidence suggests that altered microbial communities (called “microbial dysbiosis”) contribute to chronic inflammatory and systemic diseases.^[3]^

The mammalian gut mucosal immune system comprises innate intrinsic and acquired adaptive immunity. From an ecological point of view, mammals and their symbiotic microbes have co-evolved in a mutually beneficial symbiotic pattern.^[12]^ Symbiotic intestinal microorganisms enables homeostasis of immune system under normal physiological conditions. This relationship prevents symbiotic organisms from overutilizing the host’s resources or causing harm, whereas maintains host’s immune tolerance to harmless stimuli.^[13,14]^ However, disturbances in intestinal microbiota produced by alteration in microenvironment (e.g., antibiotic use, diet or geographical changes) can induce damage at host-microbiota interface as well as dysregulated immune system, which in turn induces an inflammatory response.^[15]^

As mentioned above, the intestinal microecological system consists of the normal commensal intestinal flora and the environment in which it lives (including the intestinal epithelial tissue and the intestinal mucosal immune system). The balance of intestinal microecology plays a crucial role in maintaining intestinal homeostasis. The purpose of this review is to understand the composition of the intestinal mucosal immune system, the interactions that occur between the intestinal microbiota and the mucosal immune system, and to describe the impact of altered intestinal microecology on intestinal inflammatory diseases and colorectal cancer (Table 1).

**Table 1:** The illustration of pathogenesis of intestinal diseases related to intestinal microecology.

Disease type	Pathogenesis of intestinal diseases	Reference
Inflammatory bowel disease	Microbial diversity is reduced and immune substances are produced	^[3,22,57–59]^
	Th17 and Treg cells are out of balance	^[60–62]^
	Intestinal mucosal barrier dysfunction	^[63–68]^
Colorectal cancer	Toxin production	^[8,72–76]^
	Secondary metabolite production and Changes in the tumor microenvironment	^[22,77,78]^
IBD-associated colorectal cancer	Production of inflammatory mediators	^[79,80,82,83]^
	Activation of signaling pathway	^[81,83–86]^

## 2. Composition of intestinal mucosal immune system

**Figure 1.**
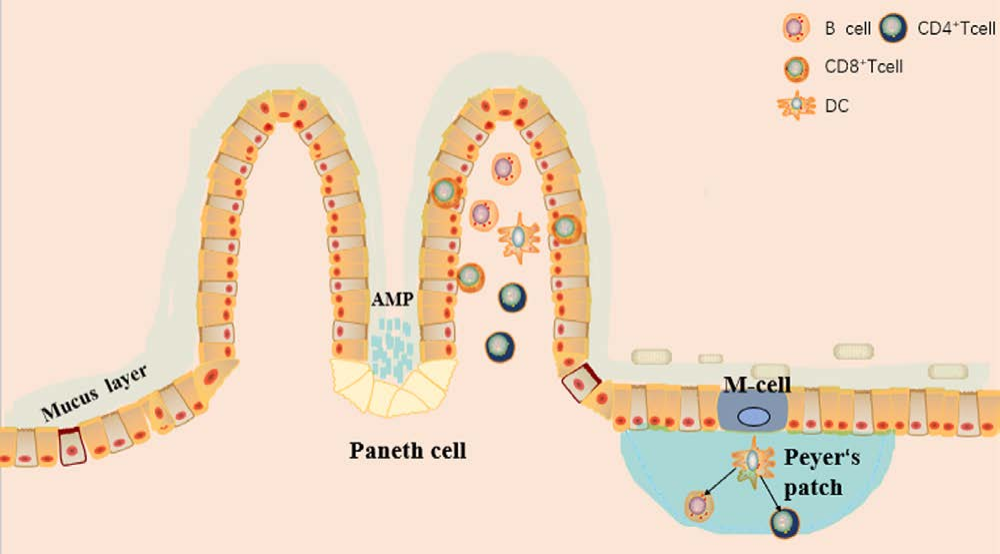
GALT organizational structure. GALT includes Piper pooled lymph nodes (PP), intraepithelial lymphocytes, and diffusely distributed lymphocytes in the lamina propria. The mucus layer on the surface of epithelial cells is the first-line physical barrier for intestinal immunity. Paneth cells are specialized epithelial cells located at the base of intestinal crypt and secrete antimicrobial peptides (AMPS) to defend against bacteria or pathogens in the intestinal cavity. Mucus together with anti-microbial peptides form a mucosal barrier can protect from invading pathogens.

Mucosa is an important component of human body’s immune system and widely distributed in respiratory, digestive and genitourinary tracts, as the major site of local immune responses. A key component of intestinal mucosa is gut-associated lymphoid tissue, which includes Piper pooled lymph nodes (PP), intraepithelial lymphocytes, and diffusely distributed lymphocytes in the lamina propria (shown in Fig. 1).^[16]^ Among them, PP is an extremely important site for initiating immune response, a dome-like structure formed by aggregation of lymphocytes that protrude into the intestinal lumen. The composition of intestinal mucosal immune system will be systematically described in the following sections.

### 2.1. Innate immunity

The mucus layer on the surface of epithelial cells is the first-line physical barrier for intestinal immunity. The production of mucus separates pathogens from the host, avoiding disruption of intestinal barrier.^[17]^ Paneth cells are specialized epithelial cells located at the base of intestinal crypt. Paneth cells secrete antimicrobial peptides (AMPS) to defend against bacteria or pathogens in the intestinal cavity, which contribute to innate defense.^[18]^ Anti-microbial peptides include alpha-defensins, lysozyme C, phospholipase, C-type agglutinin and regenerative factor 3-γ.^[18]^ These components are paramount to defend against pathogens. In addition, mucin secreted by cupped cells has a protective role in lubricating intestinal epithelial surface.^[19]^ Mucus together with anti-microbial peptides form a mucosal barrier can protect from invading pathogens.

Intestinal epithelial cells constitute the second-line physical barrier of intestinal immunity, which effectively prevent the invasion of pathogenic microorganisms. Intestinal epithelial cells are not typical immune cells, but are important components of mucosal immune system. Intestinal epithelial cells are rich in innate immune recognition receptors, to ensure homeostasis by precisely recognizing bacteria and responding to pathogens.^[20]^ Toll-like receptors (TLRs) and Nod-like receptors are the 2 most critical innate immune recognition receptors. They are well-known as pattern recognition receptors. TLRs belong to innate immune receptors that sense pathogen-associated molecular patterns (PAMPs). After sensing microbial PAMPs, TLRs are able to initiate inflammatory responses and eventually eliminate pathogenic invaders.^[21]^ In addition, intestinal epithelium includes a special type of cell, called M-cell, which cytosolically transports antigens. M-cells can endocytose and transfer antigens such as proteins from intestinal lumen directly to PP.^[22]^

During an inflammatory response, the first effector cells reaching the site of inflammation are neutrophils, which will kill and destroy pathogens in situ.^[23]^ Migration of a neutrophil depends on a variety of factors, such as chemokines and integrins.^[24]^ Upon migration to a designated location, activated neutrophils immediately produce nitric oxide, chemokines (e.g., IL-8) and pro-inflammatory factors (e.g., tumor necrosis factor-α [TNF-α] and IL-1β), leading to inflammatory response and mucosal damage.^[25]^ Dendritic cells (DCs) are required to link adaptive and innate immunity. DCs are specialized antigen-presenting cells (APCs) that can receive intestinal luminal antigens transported by M cells or acquire antigens by phagocytosis of apoptotic intestinal epithelial cells.^[26]^ DCs play an essential role in maintaining intestinal mucosal homeostasis and inducing an immune response to pathogenic bacteria. DCs exert immunomodulatory effects by producing cytokines and by inducing differentiation of primary CD4 + T lymphocytes.^[27]^

### 2.2. Adaptive immunity

A large number of T and B lymphocytes are scattered in intestinal mucosal tissue, required for adaptive immunity. Intestinal effector T cells are mainly distributed in epithelium and lamina propria, while IgA-producing B cells are mainly distributed in PP. CD8 + T cells are distributed in intestinal epithelium, while CD4+, CD8 + T cells as well as IgA + plasma cells are distributed in lamina propria^[28]^ (shown in Fig. 1). The secretion of immunoglobulins plays a crucial role in protecting intestine from microorganisms and in immune regulation. In intestinal mucosa, secretory immunoglobulin A is the largest type of immunoglobulins.^[29]^ Secretory immunoglobulin A is secreted by plasma cells (differentiated B cells) in PP and enters intestinal lumen through epithelium, where it targets microbial antigens and prevents bacterial translocation and infection.^[30]^

After stimulated by microbiota, APCs such as DCs present antigens to T cells. CD4 + T cells differentiate into Tregs and various helper T cells, such as Th17, Th1and Th2 cells, which produce IFN-γ, IL-4, and IL-17 respectively.^[31,32]^ Treg and Th17 subsets are extensively investigated, which constitute the majority of effector cells. Imbalance between these 2 subpopulations can lead to various diseases such as inflammation and autoimmune diseases.^[33]^ In addition, transfer of intestinal microbiota from mice with inflammatory bowel disease to germ-free mice could increase intestinal Th17 cells whereas decrease retinoic acid-related orphan receptor γt + Treg cells in germ-free mice compared to healthy mice.^[34]^ Thus, balance between Th17 and Treg cells plays a crucial role in maintaining immune homeostasis.

Cytokine secretion promotes the polarization of CD4 + T cells. Th17 is a subset of CD4 + T in addition to Th1 and Th2. The differentiation process of Th17 can be divided into 3 stages: differentiation, expansion, and stable maturation.^[35]^ Th17 cells secrete cytokines IL-17, IL-23, and IL-22. The pro-inflammatory mediators IL-17 and IL-23 activate nuclear factor kB related inflammatory signaling pathways whereas downregulate anti-inflammatory cytokine IL-10.^[36]^ Decreased expression of AhR in pathogenic Th17 leads to inflammation. Besides direct regulation of CD4 + T cell function by microbial metabolites, cytokines produced by DCs or macrophages, intestinal epithelial cells induced by microbial signals also regulate CD4 + T cell function.^[37,38]^

Treg cells are a subset of CD4 + T cells. Lacking of proinflammatory factors, CD4 + T cells differentiate into Treg cells, which exert negative immunomodulatory effects and maintain immune tolerance. Treg cells are involved in various immune diseases mainly by secreting anti-inflammatory cytokines (e.g., transforming growth factor-β [TGF-β] and IL-10). These cytokines can suppress effector activity of immune cells and thereby control inflammation.^[39]^ Treg cells induced by bacteria have systemic anti-inflammatory functions. Microbial signaling promotes the secretion of interleukin-1β by resident macrophages, which in turn enhances the release of granulocyte-macrophage colony-stimulating factor from intestinal ILC3 cells. Then, ILC3-derived granulocyte-macrophage colony-stimulating factor triggers the secretion of retinoic acid and IL-10 by DCs and macrophages, which in turn promotes the induction and expansion of Tregs.^[29]^

As mentioned above, secretory IgA produced by B cells in intestinal lamina propria is plays a crucial role in eliminating pathogens. Two types of helper T cells, Th17 and Tregs, are key to support IgA production in the intestine. Importantly, Tregs can assist B cells in the production of IgA.^[21]^ Transferring Tregs to mice lacking T cells will lead to differentiation of intestinal helper T cells and contribute to IgA production.^[40]^ Furthermore, Th17 cells are the primary helper cells for antigen-specific IgA production.^[41]^

## 3. Interaction between gut microbiota and immune system of intestinal microecology

The mammalian gut possesses a variety of microbiota, while human gut comprise microbial colonization from birth onward. Microbial colonization at birth leads to significant development and maturation of gut-associated lymphoid tissue in intestinal and systemic immune systems.^[42]^ Alterations in terms of number and diversity of intestinal microbiota occur during organ development. Gut microbiota has profound effects on the host, including integrity, maturation and renewal of epithelium, development of mucosal and systemic immune systems, as well as supply of energy and various vitamins.^[43]^ However, during organ development, the number and diversity of intestinal microbiota dynamically change in response to pathogenic factors, causing dysbiosis of intestinal microbiota and thus triggering a series of inflammatory reactions.

### 3.1. Gut microbiota promotes the development of immune system

The gut is an important immune organ. The intestinal microbiota can regulate the development and maturation of the host’s immune system.^[44]^ This notion has been demonstrated in germ-free mice maintained from birth into adulthood completely free of microorganisms.^[21]^ These animals exhibit a severely underdeveloped intestinal immune system compared to conventionally housed mice. For example, germ-free mice exhibit depleted intraepithelial lymphocytes, decreased size and number of Peyer and crypt spots, altered crypt structure, and diminished mucus thickness due to reduced number of cupped cells.^[45,46]^ In addition, germ-free mice (with a defective gut microbiota) present impaired immune development, characterized by immature lymphoid tissue, declined number of intestinal lymphocytes, as well as level of AMPS and IgA.^[47–49]^ Notably, reconstitution of germ-free mice with gut microbiota is sufficient to rescue these defects in and abnormalities of the immune system.^[50]^

Likewise, gut microbiota affects intestinal adaptive immunity. This has been observed in germ-free mice without intestinal bacterial colonization. The gut of a germ-free mouse has very few adaptive immune B or T cells.^[42]^ Gut microbiota contributes to physiologic development of adaptive immune B or T cells. As mentioned above, differentiated B cells produce sIgA, which acts in intestinal immunity. Undoubtedly, a decrease in B cells leads to a decrease in sIgA secretion, which negatively affects the development of intestinal immune system. In addition, gut microbiota regulates regeneration of T cells as well as distribution of T helper cells.^[51]^ For example, short-chain fatty acids produced by bacterial strains IV, XIVa and XVIII in Clostridium cluster from healthy human fecal samples can induce the differentiation and expansion of colonic Tregs. Butyrate plays a key role in this process.^[52]^ In addition, cluster IV and cluster XIVa of Clostridium perfringens could promote the induction of colonic Treg by stimulating TGF-β signaling in intestinal epithelial cells.^[42,52]^ Gur microbiota also modulate the development of intestinal Th17 cells. The abundance of Th17 cells in the intestinal mucosa was significantly reduced in germ-free or antimicrobial-treated mice.^[51]^ Furthermore, microorganisms with adhesion properties to intestinal epithelial cells, such as Escherichia coli O157 and Citrobacter, could induce Th17 cells.^[53]^

### 3.2. Dysbiosis of intestinal flora triggers inflammatory response

Gut bacteria actively interact with the host’s immune system, undoubtedly, dysregulation of balances/interactions can lead to inflammation-related diseases.^[10,11]^ Accumulating evidence suggests that altered microbial communities (called “microbial dysbiosis”) and intestinal barrier damage contribute to pathogenesis of chronic inflammatory and systemic diseases.^[3]^ Microbial community dysbiosis is characterized by changes in microbial populations, diversity, distribution or quantity.^[54]^ More specifically, microbial dysbiosis causes alterations in intestinal epithelial mucosa and promotes inflammation by modulating cytokine activity. Dysbiosis enables bacterial penetration into (otherwise almost sterile) inner layer of intestinal mucosa, which introduces microorganisms into contact with epithelial cells, and thus triggering inflammatory responses.^[55]^

Generally, inflammatory response is mediated by TLRs, which play a key role in host’s recognition of microorganisms. Specifically, TLR4 and TLR5 is involved in recognizing bacterial lipopolysaccharide and bacterial flagellin, respectively.^[56]^ As a result, inflammatory responses mediated by TLRs initiate chemotaxis and migration of neutrophils to inflammation site, to produce a series of inflammatory factors. Immediately afterwards, M cells present pathogens to APCs, in particular DCs, initiating innate and adaptive immune responses. Thus, M cell is an unique component of intestinal mucosal entity.

## 4. Intestinal micro-ecological alterations induce intestinal disease

### 4.1. Intestinal microecology and inflammatory bowel disease

**Figure 2.**
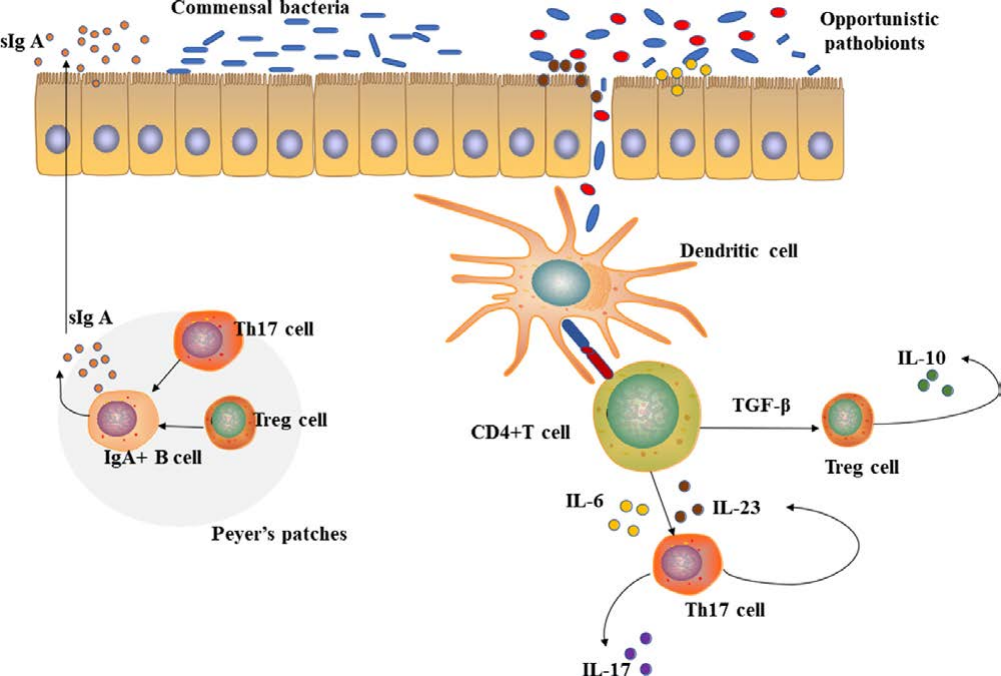
Intestinal microecological changes lead to intestinal inflammation. The imbalance of intestinal flora or the destruction of intestinal barrier will lead to the change of intestinal microecology and the change of intestinal immune system. The pro-inflammatory factors secreted by Th1 and Th17 cells are unbalanced with the anti-inflammatory factors secreted by Treg cells. Two types of helper T cells, Tregs and Th17 cells, can assist B cells to produce secretory IgA to help eliminate pathogens in the intestine.

In order to safeguard intestinal homeostasis, a balance is maintained between intestinal microorganisms and intestinal mucosal immune system. However, if this balance is broken, dysfunction of immune system is inevitable, and thus intestinal disorders, such as inflammatory bowel disease (IBD) (shown in Fig. 2). IBDs, comprising mainly ulcerative colitis and Crohn disease (CD), are multifactorial diseased of unknown etiology, which can cause intestinal inflammation by triggering abnormal immune responses.^[22]^

Alterations in microbial community diversity are common in IBD, characterized by reduction in commensal bacteria, such as Firmicutes and Bacteroidetes, whereas enrichment in harmful bacteria, such as Proteus and actinomycetes.^[57]^ Microbial abundance in IBD patients is reduced by an average of 25% compared to healthy individuals.^[3,58,59]^ Accordingly, gut microbiota in IBD cannot adapt to environmental changes or resist natural interference because of reduction in microbial diversity, and thus producing immunogenic substances. In addition, Th17/Treg imbalance is a hallmark of IBD pathogenesis, resulting in a series of abnormal immune responses. Th17/Treg cells are interconnected through differentiation and functional inhibition. They share a common signaling pathway mediated by TGF-β. It has been declared that naive CD4 + T cells differentiate into Th17 cells in the presence of IL-6, IL-21 or TGF-β; however, in the absence of pro-inflammatory cytokines, naive CD4 + T cells differentiate into Treg cells.^[60]^ If this balance is disturbed, autoimmune diseases can be expected, including IBD. In the intestinal mucosa of patients with IBD, Th17 infiltration and IL-17 (a cytokine specifically secreted by Th17 cells) are increased compared to healthy controls.^[61]^ In contrast to Th17, Treg cells have a negative regulatory effect on autoimmune diseases and intestinal inflammation. In a ulcerative colitis mouse model, Treg cells were decreased in peripheral blood.^[62]^

When physical barrier is destroyed, immunogenic substances can pass through the intestinal wall and cause damage.^[22]^ The intestinal barrier is dependent on serial interactions between mucus layer, IgA, AMPs and intercellular tight junctions (TJs).^[63]^ The mucus layer stabilizes intestinal lining. The most obvious pathological feature of human IBD is a decrease in mucin secretion due to gradually depleted Cup-shaped cells. For example, mice lacking mucin MUC2 develop spontaneous colitis.^[64]^ In addition, intestinal epithelial cells secrete AMPs to regulate luminal microbial colonization and infiltration of epithelial cells.^[65]^ Notably, intercellular TJs are the main determinants of intestinal physical barrier. TJs are assembled from a variety of proteins located near apical epithelial cell membrane.^[63]^ Claudin proteins are a critical component of TJs and a paramount physical defense against pathogen invasion. Abnormal expression of Claudin can lead to reduced adhesion, structural damage, and impaired function of epithelial and endothelial cells.^[66]^ Importantly, inducible intestinal Cldn7 knockout could cause disruption of the intestinal mucosal barrier in mice.^[67]^ Intestinal epithelial-specific knockdown of Cldn-7 increases susceptibility to DSS-induced colitis.^[68]^ In addition, mice developed histopathological features of CD at 3 months of age due to endogenous E-calcineurin deficiency.^[3]^ Collectively, disruption of intestinal barrier exacerbates intestinal inflammation.

Although IBD has unknown etiology, emerging evidence suggests that genetic susceptibility, defects in mucosal barrier function, immune stimulation, as well as alterations in microbial composition and function of intestinal environment contribute to its pathogenesis.^[69,70]^ Under normal conditions, intestinal microbiota facilitates physiologic development of immune system and maintains intestinal homeostasis. However, when the balance between intestinal microbiota and mucosal immune system is damaged, a series of inflammatory diseases develop, such as IBD, systemic lupus erythematosus, rheumatoid arthritis and ankylosing spondylitis.^[22]^

### 4.2. Intestinal microecology and colorectal

Intestinal microecological disorder is a prominent feature of colorectal cancer, and one study showed^[71]^ that colorectal cancer patients had significantly lower abundance and diversity of intestinal flora. However, the specific mechanism of the role of intestinal flora in the development of colorectal cancer is unclear, and according to available studies, it mainly includes 3 aspects: (1) causing intestinal epithelial cell damage through toxins; (2) causing metabolic disorders in the body through secondary metabolites; and (3) interacting with the intestinal immune system to alter the tumor microenvironment and promote tumor immunosuppression.

#### 4.2.1. Toxin production by intestinal flora promotes colorectal carcinogenesis.

Certain bacteria in the intestine, such as *Escherichia coli, Campylobacter jejuni, Fusobacterium nucleatum*, and *Bacteroides fragilis*, can express toxin proteins that activate downstream signaling pathways, causing intracellular reactive oxygen species accumulation and DNA damage, leading to colorectal carcinogenesis.

*F. nucleatum* is a bacterium closely associated with periodontal disease. It has been shown^[72]^ that *F. nucleatum* promotes CRC growth through its unique FadA adhesin, which binds to E-cadherin (CDH1) and activates Wnt/β-catenin signaling to promote cell proliferation and DNA damage, inducing inflammatory responses and tumorigenesis. In addition, it has also been shown^[73]^ that *F. nucleatum* promotes CRC metastasis via miR-1322/CCL20 and M2 polarization. Epidemiological studies have shown that patients with colorectal cancer have a significantly higher abundance of ETBF compared to normal patients.^[74]^ ETBF can promote tumorigenesis by releasing a genotoxic compound, *Bifidobacterium fragilis* toxin, which activates the NF κB signaling pathway and the Wnt/β catenin pathway, causing excessive proliferation of intestinal epithelial cells and inducing an intestinal inflammatory response that promotes colorectal carcinogenesis.^[75]^ *Campylobacter jejuni* can bind and invade intestinal epithelial cells. It has been shown that the cytolethal distending toxin secreted by *C. jejuni* can cause DNA double-strand breaks in host cells, leading to cell cycle arrest and thus promoting colorectal carcinogenesis.^[76]^ In addition, *Campylobacter jejuni* 81–176 activates the NF κB signaling pathway, promotes IL 6 expression, and exacerbates the intestinal inflammatory response.^[8]^

#### 4.2.2. Secondary metabolite production by intestinal flora affects colorectal cancer progression.

Intestinal flora can not only directly adhere to and invade intestinal epithelial cells, but also influence colorectal carcinogenesis through the production of secondary metabolites, such as short-chain fatty acids and secondary bile acids. They modulate intestinal barrier function and affect immune cell differentiation and function, which in turn affects tumor progression. Short-chain fatty acids, which play an important role in the prevention and inhibition of colorectal cancer, are a class of substances produced by fermentation of dietary fiber by symbiotic bacteria in the intestine, including propionate and butyrate, which have anti-inflammatory and anti-tumor effects. For example, *Clostridium butyricum* is a butyrate-producing probiotic that inhibits the development of intestinal tumors by regulating Wnt signaling and intestinal microbiota.^[77]^ At the intestinal level, butyrate improves mucosal inflammation and oxidative status, strengthens epithelial defense barriers, and regulates visceral sensitivity and intestinal motility.^[78]^

The intestinal flora plays an important role in the nutrient absorption and digestive metabolism of the host. It can affect the health and homeostasis of the gut through the production of metabolites. Therefore, the use of diet to modulate the intestinal flora and thus interfere with the intestinal disease process may provide a new direction for colorectal cancer treatment, but the exact mechanisms remain to be explored.

#### 4.2.3. Intestinal flora alter the tumor microenvironment to promote the development of colorectal cancer.

In addition to affecting colorectal cancer through its own production of toxins and metabolites, intestinal flora can also promote the development of colorectal cancer by altering the tumor microenvironment. As mentioned earlier, intestinal flora dysbiosis induces intestinal immune response and alteration of intestinal microecology promotes colorectal cancer development. For example, *Fusobacterium nucleatum*, mentioned above, can bind to immune cell surface receptors through adhesion factors, activate immunosuppressive signals, recruit tumor-infiltrating myeloid cells, alter the tumor microenvironment and thus promote tumor growth and infiltration.^[21]^ Intestinal flora promote disease development by inducing immune responses, altering the ratio of immune cells, and establishing a microenvironment suitable for tumor cell survival. This suggests that targeting specific intestinal flora may enhance the effect of tumor immunotherapy and slow down the progression of colorectal cancer.

### 4.3. Intestinal microecology and IBD-associated colorectal cancer

**Figure 3.**
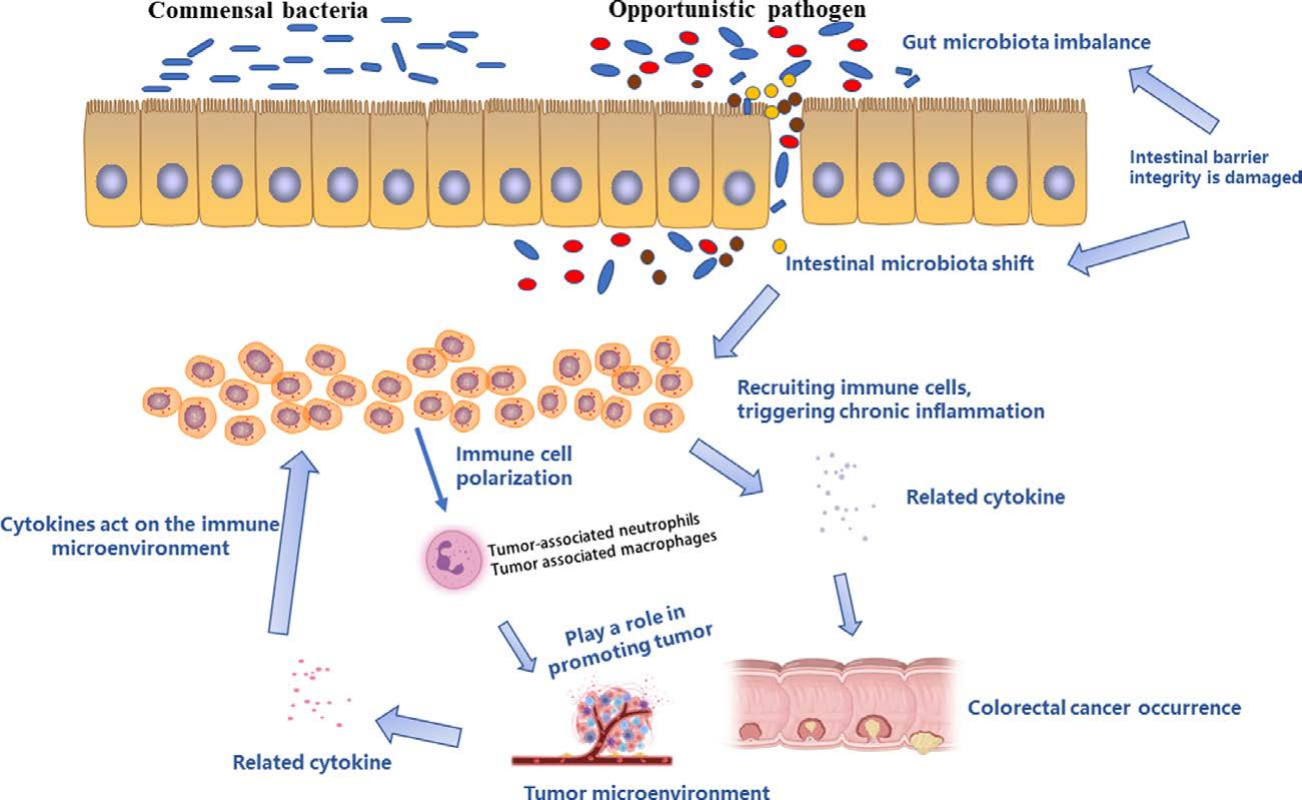
Correlation between intestinal microecology and colorectal cancer. The integrity of the colon epithelium is damaged, resulting in the imbalance of the intestinal microecology and the displacement of the intestinal microflora, which in turn recruits immune cells to promote the inflammatory cascade, forming chronic intestinal inflammation and promoting the occurrence of colorectal cancer. After the occurrence of colorectal cancer, tumor cells and tumor-associated macrophages release chemokines and cytokines, under which immune cells plan to further affect the microenvironment of colorectal cancer, promote tumor growth, invasion, metastasis, angiogenesis, and inhibit immunity.

Chronic intestinal inflammation is one of the most common risk factors for colorectal cancer. Compared to the general population, people with IBD, including ulcerative colitis and CD, are at a higher risk of developing colitis-associated colorectal cancer.^[79,80]^ Studies have identified key genes and molecular pathways in the progression of CD and colorectal cancer.^[81]^ During inflammation, recruitment of the above-mentioned immune cells leads to the secretion of highly genotoxic oxygen/nitrogen active substance,^[82]^ pro-inflammatory cytokines such as interleukin (IL-6), IL-8, IL-1β, TNF-α, and growth factors.^[83]^ The production of these mediators is mediated by several major signaling pathways, including nuclear factor kB, signal transduction and transcriptional activator 3, PI3K/AKT, and cyclocoxidase-2/prostaglandin E2. They are involved in many processes such as proliferation, angiogenesis, invasion, metastasis and recruitment of inflammatory mediators.^[83]^ In addition to the above cytokines and immune cell involvement, the development process of IBD-CRC may also be due to chromosome and microsatellite instability, triggered by well-defined pathways (Wnt pathway, CIMP pathway), resulting in mucosal dysplasia.^[84]^ These studies all suggest that chronic inflammation triggers and drives tumorigenesis,^[85]^ resulting in an “inflammatory - dysplastic carcinoma” sequence rather than the “adenomatous – sequence” classically described in sporadic colorectal cancer.^[86]^ (shown in Fig. 3).

As we all know, bacteria can be oligomerized with TLRs, retinoic acid-inducible gene I-like receptors and nucleotide binding by PAMPs domain-receptors communicate and trigger immune responses.^[85]^ NF-kB can be activated by TLR and TNF-α to induce the transcription of multiple tumor-generating genes such as cyclocoxidase-2, and then lead to the destruction of intestinal barriers such as apoptosis of intestinal epithelial cells through the tumor-suppressing p53 pathway,^[86]^ resulting in microbial translocation. Some studies have shown that bacteria such as *E. coli*, ETBF, and Fusobacterium nuclei are involved in chronic inflammation and cancer development in patients with IBD.^[87,88]^

Popov et al^[87]^ proposed 3 main theories on the involvement of intestinal flora in IBD-related CRC development: The alpha-bug hypothesis, driver-passenger hypothesis, and common ground hypothesis. In the alpha-bug hypothesis, a single bacterium (usually ETBF) is thought to be the driver of cancer. The driver-passenger hypothesis is similar, but after the first bacterial attack, other opportunistic bacteria begin to grow and lead to the development of cancer. Finally, in the common basis hypothesis, endogenous and exogenous factors form a “leaky gut” that allows bacteria to enter submucosal tissues, leading to chronic inflammation and subsequent cancer.

## 5. Conclusion

In summary, coordinated formation of host immunity by intestinal microbiota would be essential to maintain intestinal homeostasis and to inhibit inflammation. However, alterations in gut microecology, such as impaired interactions between mucosal immune system and gut microbiota, or disruptions in gut barrier function, inflammatory diseases will develop. This process highlights the importance of gut microecology in inflammatory diseases.

In addition, intestinal microecology, as an important component of the human body, plays an equally important role in the development and treatment of colorectal cancer. Some intestinal flora, through secretion of toxins, production of secondary metabolites and alteration of tumor immune microenvironment, affect the development of colorectal cancer. The role and mechanism of intestinal microecology in intestinal diseases and health will be continuously discovered and confirmed as related research progresses.

## 6. Search strategy

References were searched in PubMed, Web of science, to analyze the role of intestinal microecology in intestinal inflammatory bowel disease and colorectal cancer from the time of library construction to December 2022. Most of the references are from the last ten years. English search terms included (1) “colorectal cancer” OR “colon cancer” OR “rectal cancer” OR “IBD” OR “ Inflammatory bowel disease”; (2) “microbiota” OR “intestinal microbiota” OR “intestinal microbiology”; (3) “intestinal immunity” OR “intestinal mucosal immune system.”

## Author contributions

**Conceptualization:** Huimin Li, Wang Kun.

**Formal analysis:** Huimin Li, Yin Liu.

**Supervision:** Lei Ding.

**Writing – original draft:** Huimin Li.

**Writing – review & editing:** Mengdi Hao, Xiaoqing Liang, Dajin Yuan.
